# Physicians' Self-Assessed Empathy and Patients' Perceptions of Physicians' Empathy: Validation of the Greek Jefferson Scale of Patient Perception of Physician Empathy

**DOI:** 10.1155/2020/9379756

**Published:** 2020-02-12

**Authors:** Vasiliki Katsari, Athina Tyritidou, Philippe-Richard Domeyer

**Affiliations:** School of Social Sciences, Hellenic Open University, Parodos Aristotelous 18, Patras 26 335, Greece

## Abstract

**Aims:**

This study aims to (i) translate, culturally adapt, and validate the Jefferson Scale of Patient Perception of Physician Empathy questionnaire for the Greek population (Gr-JSPPPE) and (ii) estimate physicians' self-assessed empathy and patients' perceptions of physicians' empathy, investigate their relationship, and assess their predictors.

**Methods:**

A total of 189 patients and 17 physicians from an internal medicine clinic took part in the study. A composite questionnaire was administered to the patients, consisting of (1) sociodemographic items, (2) hospitalization-related questions, (3) the Zung Self-Rating Anxiety Scale, (4) the Patient Health Questionnaire (PHQ-9), (5) the EQ-5D-5L Questionnaire, (6) the Gr-JSPPPE, and (7) the Visual Analog Scale for pain. The physicians' composite questionnaire comprised (1) sociodemographic items, (2) the EQ-5D-5L questionnaire, and (3) the Toronto Composite Empathy Scale (TCES). Exploratory and confirmatory factor analyses were conducted to assess the psychometric properties of the Gr-JS PPPE. Univariate comparisons were performed between (a) empathy measures and (b) sociodemographic and health-related measures of both groups; multivariate regression analysis for the Gr-JSPPPE adjusting for baseline confounders was executed.

**Results:**

Statistically significant negative correlations were found between the Gr-JSPPPE mean score and the TCES personal/cognitive, professional/cognitive, and professional/emotional subscales. Female sex, being married, duration of employment in current post, and physicians' EQ-5D index score emerged as important predictors of increased physician empathy. Patients' EQ-VAS “thermometer” scale was significantly associated with the Gr-JSPPPE total score at the multivariate level.

**Conclusion:**

The Gr-JSPPPE is a psychometrically sound tool to assess patient perceptions of physician empathy. Physician empathy assessed by the self-reported scale is inversely associated with patient perceptions.

## 1. Introduction

Empathy is considered a prerequisite for a successful physician-patient relationship, an integral part of high-quality patient-centered healthcare, and is regarded as probably the most robust evidence of the humanitarian side of medicine [1–3]. Although the notion of empathy is not clearly and universally defined in the international literature [1, 2, 4–11], some of its key components can be unanimously recognized, namely, the physician's potential (a) to acknowledge the inner experiences as well as emotional state of the patient, (b) to communicate this acknowledgment to the patient, and (c) to adopt a positive and therapeutic behavior [12].

Empathic engagement in patient care seems to exert positive influences on both patients and physicians. It has been linked with decreased patient pain [13, 14] and anxiety [15], increased patient satisfaction [12, 16–23], increased adherence to treatment [18, 24], and improved clinical outcomes [12, 21, 24–30]. In addition, empathic physicians demonstrate a higher level of well-being [31–33], achieve higher ratings of clinical skills [34], suffer from lower levels of burnout [33], and are at decreased risk of medical malpractice [35–37]. Strikingly, through the adoption of patient-centered communication, physician empathy has even been associated with lower diagnostic test expenditures [38].

Physician empathy may be assessed either by (a) self-rating, denoting the completion of standardized questionnaires by the physicians themselves, (b) patient-rating, signifying the assessment of physician empathy by their patients as experienced by them, and (c) third-person rating, indicating the evaluation of physician empathy towards their patients by an observer. A systematic review by Hemmerdinger et al. [39] identified a total of 36 instruments. Among 14 first-person assessments, only 6 of them had evidence of either reliability or internal consistency testing, whereas among 5-second person assessments, only the Consultation And Relational Empathy (CARE) scale demonstrated such evidence. Lately, evidence of validity and reliability for the Jefferson Scale of Patient Perception of Physician Empathy (JSPPPE), the shortest available instrument (5 items), was published [40]. This tool has attracted considerable notice and is now available in eleven languages [40].

Although it seems rational that the assessment of physician's empathy should also include the patients' perspective, to the author's knowledge, only four studies have explored the putative correlation between physicians' empathy as perceived by patients and as assessed by themselves, with the use of validated instruments [3, 41–43]. In all these studies, physicians and patients filled out the Jefferson Scale of Physician's Empathy (JSPE) and the JSPPPE, respectively. Further, in the most recent and largest study [43], the physicians additionally completed the International Reactivity Index scale and the patients were also administered the CARE scale. Interestingly, among these four studies, only one showed a positive and significant correlation [3]. As a result, the degree to which self-assessed empathy coincides with patients' views remains an open field. In addition, there is complete absence of any validated tool gathering patient feedback on physicians' empathy in the Greek literature.

The current study aims to (i) translate, culturally adapt, and validate the JSPPPE for the Greek population (Gr-JSPPPE) and (ii) estimate physicians' self-assessed empathy and its perception by patients, investigate their relationship, and assess their predictors.

## 2. Methods

### 2.1. Study Sample

The study was conducted from March to May 2018 at the Third Pathological Clinic of the “Papageorgiou” General Hospital in Thessaloniki, Greece. Its sample included two distinct groups. Group A consisted of all patients admitted to the clinic during the above period and group B comprised the patients' physicians. Patients with severe mental illness (dementia, aphasia, and uncontrolled psychosis) or experiencing any difficulty with the Greek language that would compromise their reading comprehension were excluded from the study.

### 2.2. Questionnaire Structure

#### 2.2.1. Group A

The composite study questionnaire comprised the following:

1. Sociodemographic questions, i.e., age, sex, marital status, number of household members, education, professional status (employed, unemployed, student, and housewife), and net personal monthly income.
2. Hospitalization-related questions including the length of stay and diagnosis, classified according to the Greek Diagnosis-Related Groups (DRGs).
3. The Zung Self-Rating Anxiety Scale (SAS), which is a 20-item self-rating tool assessing anxiety levels according to 4 symptom groups: cognitive, autonomic, motor, and central nervous system, such as “I feel more nervous and anxious than usual” (item 1). The answers, evaluating symptom frequency, are given on a 4-point Likert-type scale (“a little of the time,” “some of the time,” “good part of the time,” and “most of the time”). The total score range is 20 to 80; higher scores denote increased anxiety [44].
4. The Patient Health Questionnaire (PHQ-9), which is a 9-item self-administered tool using a 4-point Likert-type scale assessing the degree of depression according to symptom frequency (“not at all,” “several days,” “more than half the days,” and “nearly every day”), such as “little interest or pleasure in doing things” (item 1). The total score range is 0–27; a score of 10 has been suggested as a cutoff for the existence of depression [45].
5. The EQ-5D-5L questionnaire, which is a generic instrument measuring the quality of life by using 5 degrees of problem severity (none, slight, moderate, severe, and extreme) for each of the 5 dimensions (mobility, self-care, usual activities, pain/discomfort, and anxiety/depression) [46]. According to patients' responses, the EQ-index values were calculated; the time trade-off (TTO) valuation technique using published scoring functions for the EQ-5D-3L was adopted [47]; the crosswalk link function was subsequently chosen to compute index values for the EQ-5D-5L [48]. In addition, EQ-5D comprises a visual analog scale for recording patients' assessment of their own health status on a 0–100 scale, named EQ-5D VAS “thermometer” [49].
6. The Jefferson Scale of Patient Perceptions of Physician Empathy (JSPPPE), which is a 5-item tool rating the patient's understanding of his/her physician's degree of empathic behavior. Every answer is recorded on a 7-point Likert-type scale (ranging from 1 = strongly disagree to 7 = strongly agree) [41, 42]. The scale score is generated by adding individual item scores; a higher score is correlated with a more empathic perception of the physician by the patient. Psychometrics of the original [40], a French [50], and a modified Spanish version [51] of the JSPPPE have been studied; evidence of validity and reliability for all versions has been documented.
7. The Visual Analog Scale (VAS) for pain, a unidimensional single-item scale assessing the patients' level of pain, consisting of a straight line with the endpoints denoting two extremes, i.e., “no pain” and “pain as bad as it could be” [52].

#### 2.2.2. Group B

The composite study questionnaire consisted of

1. Sociodemographic questions, i.e., age, sex, marital status, number of household members, duration of employment in current post and total years of work experience, average duration of night sleep, and net personal monthly income.
2. The EQ-5D-5L questionnaire.
3. The Toronto Composite Empathy Scale (TCES), consisting of 52 questions evaluating empathy's cognitive as well as emotional aspects, in personal as well as professional settings, resulting in four subscales i.e., Personal Emotional (such as “I find it hard to *feel sorry* when they are having problems”), Professional Emotional (such as “I find it hard to *feel sorry for my patients* when they are having problems”), Personal Cognitive (such as “I try to understand *people better by imaging* how things look from their perspective”), and Professional Cognitive (such as “I try to understand *my patients better by imagining* how things look from their perspective”), each encompassing 13 questions [53]. Every question is answered on a 6-point Likert-type scale, according to the frequency of occurrence of thoughts and behaviors (“all of the time,” “most of the time,” “slightly more than half the time,” “slightly less than half the time,” “some of the time,” and “at no time”) [53]. Every subscale's score may vary between 13 and 78, with higher scores denoting increased empathy. The scale has been translated into Greek and has shown evidence of validity and reliability [54].

### 2.3. Translation and Cultural Adaptation of the JSPPPE

Written permission to translate the original JSPPPE version to Greek for research purposes was obtained from Kane et al. [41]. The translation procedure was in line with the guidelines concerning the cross-cultural adaptation of self-administered questionnaires [55].

The JSPPPE was initially translated into Greek by two independent experienced research and clinical physicians that were native Greek speakers with advanced knowledge of English (forward translation). A reconciliation meeting took place with the participation of a third independent reviewer, to create a consensus version (reconciliated Greek version). Two bilingual physicians, who had no prior knowledge of the instrument, retranslated the reconciliated version to the original language (back translation). The back translations were then compared and inconsistencies were addressed until consensus was reached regarding a final back translation document. This version was subsequently compared with the original English version of the JSPPPE, for final confirmation of the linguistic accuracy. In line with the usual cultural adaptation procedure, the instrument was handed over to five registered physicians to verify the clarity of each item, to ascertain its face validity. The last step consisted of sending the questionnaire to ten academics and health experts to highlight irrelevant, ambiguous, or problematic items, therefore ensuring its content validity. No necessary modifications of the questionnaire emerged from these two final steps.

### 2.4. Questionnaire Administration

Patients were administered the paper-based composite questionnaire on the last day of their hospitalization by the researcher (AT), who was not involved in their treatment. Patients were reassured that their treating physician would remain blind to their responses and that their medical treatment would remain unaffected. The same researcher administered the composite questionnaire to the treating physicians. Anonymity and confidentiality were guaranteed for all participants.

The study was approved by the Institutional Review Board. Patients and physicians unanimously consented to participate in the study and gave their written informed consent. No compensation was provided to the participants.

### 2.5. Statistical Analysis

To begin, the study sample was randomly split into two halves. Exploratory factor analysis (EFA) was conducted on the first half for the creation of multi-item scales for the Gr-JSPPPE. Confirmatory factor analysis (CFA) was carried out in the second half to assess the model fit.

#### 2.5.1. Exploratory Factor Analysis

Sample size exceeded the minimum 5 : 1 subjects-to-variable ratio adequate for EFA [56, 57]. The normality of the data was checked, as the multivariate normal distribution is a prerequisite for factor analysis. Although multivariate normality was violated, in the case of large samples, a good approximation of the normal distribution can be attained with the application of the multivariate Central Limit Theorem, therefore permitting the use of factor analysis [58]. Bartlett's test for sphericity was used to test the suitability of the variables for structure detection. The Kaiser-Meyer-Olkin test, measuring sampling adequacy for both individual model variables and the complete model, was also computed; a cutoff value of 0.5 is warranted for satisfactory results [59], with levels between 0.8 and 0.9 being regarded as great [60].

For the EFA, the principal axis factors method was used due to the assumption for multivariate normality being violated [61]. Two criteria were adopted to decide on the number of factors to retain: Kaiser's criterion, suggesting that only factors with eigenvalues higher than one must be retained, and the scree plot.

#### 2.5.2. Confirmatory Factor Analysis

During CFA, the fit of the data to the model was evaluated. The Maximum Likelihood (ML) estimation method used in CFA is based on the multivariate normality assumption, which was found to be violated. In line with previous work [62], the Bootstrap Maximum Likelihood Estimator was adopted instead for estimating estimation standard errors and confidence intervals [63]. According to the suggestion by Nevitt and Hancock [64], the number of bootstrap samples was set at 250. The bias-corrected and accelerated bootstrap confidence interval was set at the 95% level. Fit indices assessed included the following: (i) the Bollen–Stine bootstrap *p* statistic that was adopted instead of *χ*2 *p*, where appropriate [65]; a cutoff of *p* > 0.05 was regarded to designate adequate model fit, (ii) the Standardized Root Mean Square Residual (SRMR), whose values lower than 0.08 are deemed acceptable [58], (iii) the goodness-of-fit index (GFI) and adjusted goodness-of-fit index (AGFI), whose values of 0.95 and 0.90 indicate well-fitting models, respectively [66], (iv) the normed fit index (NFI), with values exceeding 0.95 being considered as a good fit [58], and (v) the Tucker Lewis Index (TLI) and the Comparative Fit Index (CFI); cutoff values of 0.90 and 0.95 correspond to acceptably and well-fitting models, respectively [67, 68]. The squared multiple correlations (R2) were also computed for each measured model parameter [69]. The model fit was ameliorated by assessing modification indices (MIs) during the post hoc analyses [70].

#### 2.5.3. Internal Consistency Reliability

Cronbach's alpha coefficient was adopted as an indicator of internal consistency. Other reliability statistics used were the range of interitem correlations, the corrected item-scale correlations, and Cronbach's alpha for a measure if a single item is deleted (corrected Cronbach's alpha).

#### 2.5.4. Test-Retest Reliability

To compute test-retest reliability, 30 patients filled out the instruments twice, i.e., 24 hours after admission to the clinic and the last day of their hospitalization, provided that at least one week and no more than two weeks had elapsed. The sensitivity of the scale and its individual items to external factors was estimated by assessing test-retest reliability using intraclass correlation coefficients (ICC), which were computed using a two-way mixed-effects model [71, 72].

#### 2.5.5. Ceiling and Floor Effects

Ceiling and floor effects were computed, whose presence weakens the scale's content validity; their existence indicates that several subjects' scores consistently cluster towards the best and worst level of scores, respectively.

Data were analyzed with IBM SPSS Statistics version 24 and IBM SPSS AMOS version 25 (Chicago, IL, USA). The mean (standard deviation) and median (interquartile range) were computed in the case continuous variables; absolute and relative frequencies (*n*; %) were reported in the case of categorical variables. Interitem and item-scale correlations were calculated using Pearson's correlation coefficient (*r*). In group A, univariate analyses were performed to investigate associations between sociodemographic characteristics, health-related measures, and the Gr-JSPPPE. The parametric independent-samples *T*-test and Analysis of Variance (ANOVA) were used for evaluating differences between groups; Pearson's correlation coefficient (*r*) was employed to calculate the correlation between continuous variables and the Gr-JSPPPE. Predictors significant on univariate analyses were entered in the multivariate linear regression analysis. In group B, univariate analyses were carried out to highlight associations between sociodemographic characteristics, health-related measures, and the TCES subscales. Due to small sample size, nonparametric tests were used, i.e., the Mann-Witney *U* test and the Kruskal-Wallis test to test the statistical significance of group differences and Spearman's correlation coefficient (*r*) to evaluate the strength and direction of association between continuous variables and the TCES subscales. The latter was also used to estimate the magnitude of association between the Gr-JSPPPE and TCES subscales. For the same reason, multivariate analyses were not undertaken in this group. The statistical significance level was set at *p* < 0.05.

## 3. Results

### 3.1. Exploratory Factor Analysis

The Kaiser-Meyer-Olkin Measure of Sampling Adequacy (KMO = 0.752) and Bartlett's test of sphericity (*χ*^2^(10) = 421.468, *p* < 0.001) suggested that data were adequate for conducting an exploratory factor analysis. The factor analysis yielded only one factor, which explained 72.52% of the entire variance (Table 1).

### 3.2. Confirmatory Factor Analysis

**Figure 1:**
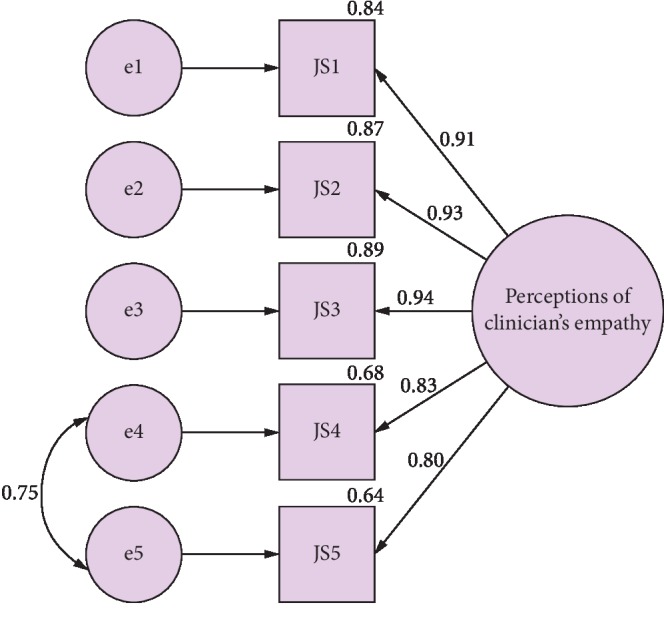
Path diagram for the confirmatory factor analysis with standardized regression estimates and squared multiple correlations.

A confirmatory factor analysis was performed to assess the extent to which the proposed 5-item scale fit the data. Unacceptable fit results emerged: Bollen–Stine bootstrap *p*=0.004, SRMR = 0.057, GFI = 0.760, AGFI = 0.279, NFI = 0.845, TLI = 0.701, and CFI = 0.851. The deletion of the JS5 item with the lowest factor loading and a very high correlation with JS4 did not improve the fit results (data not shown). After examining the MIs, the error terms of JS4 and JS5 were covaried, resulting in a significantly improved model fit: Bollen–Stine bootstrap *p*=0.291, SRMR = 0.020, GFI = 0.936, AGFI = 0.761, NFI = 0.968, TLI = 0.936, and CFI = 0.974 (Figure 1).

### 3.3. Internal Consistency Reliability

Cronbach's alpha coefficient for the Gr-JSPPPE scale was 0.935, denoting excellent internal consistency. The range of interitem correlations was 0.624–0.912; the only correlation above 0.85 was between JS4 and JS5. The corrected item-total correlations and the corrected Cronbach's alpha values if a single item is deleted are presented in Table 2.

### 3.4. Test-Retest Reliability

The test-retest reliability analysis provided excellent results, with all ICC values ranging between 0.848 and 0.958 (Table 3).

### 3.5. Ceiling and Floor Effects

In the case of ceiling effects, the percentages of observations corresponding to the best score category for the five items and the total Gr-JSPPPE score were 25.4%, 20.6%, 21.7%, 54.5%, 63.0%, and 14.3%, respectively. The percentages in the case of flooring effects were 3.2%, 7.4%, 6.9%, 2.6%, 1.1%, and 0.5%, respectively. Only the JS4 and JS5 items presented a considerable ceiling effect.

### 3.6. Participants' Characteristics and Health-Related Measures

A total of 189 patients and 17 physicians completed the composite questionnaires; their sociodemographic characteristics are detailed in Table 4. The average duration of the patients' hospitalization was 5.7 (3.7) days. The commonest diagnoses for hospitalization, whose cumulative frequency exceeded 50%, were (a) infectious diseases (*n* = 44; 23.2%), (b) anemias (*n* = 27; 14.3%), (c) gastrointestinal hemorrhages (*n* = 17; 9.0%), and (d) strokes (*n* = 16; 8.4%). The scores of the Gr-JSPPPE are presented in Tables 2 and 3, according to which the JS4 and JS5 items showed the highest mean and median rank scores. The scores of the participants' other health-related measures are illustrated in Table 5.

Statistically significant negative correlations were found between the Gr-JSPPPE total score and the TCES personal/cognitive (*r* = −0.244, *p*=0.001), professional/cognitive (*r* = −0.225, *p*=0.002), and professional/emotional (*r* = −0.174, *p*=0.017) subscales. However, the correlation between the Gr-JSPPPE total score and the TCES personal/emotional subscale was not statistically significant (*r* = −0.118, *p*=0.107). The following correlations were revealed during the univariate analyses of TCES subscales with group's B sociodemographic and health-related measures subscales: (i) TCES personal/cognitive subscale was positively associated with female sex (*p*=0.046) and being married (*p*=0.045), (ii) TCES professional/cognitive subscale was positively associated with duration of employment in current post (*p*=0.025) and marginally with female sex (*p*=0.059), and (iii) TCES professional/emotional subscale was negatively associated with the physicians' EQ-5D index score (*p*=0.005). At the multivariate level, only the group's A EQ-VAS “thermometer” scale demonstrated a significant association with the Gr-JSPPPE total score (*β* = 0.043, *p*=0.028).

## 4. Discussion

In this study, the JSPPPE was translated and validated for the Greek population for the first time. During the exploratory factor analysis, a unidimensional scale emerged with high factor loadings, in line with previous work [40–51]. Furthermore, this is the first study to perform a confirmatory factor analysis on the 5-item 7-point Likert-type JSPPPE scale, as the only other available study used one 5-item version and one modified 6-item version of the instrument, both with a 5-point Likert-type answer scale [51]. In that study, the possible violation of the multivariate normality assumption along with the small degrees of freedom may have explained the inflated RMSEA for both instrument versions (0.098 and 0.158, respectively) [73]. For these reasons, we did not compute the chi-square *p* statistic and RMSEA index but decided to assess the Bollen–Stine bootstrap *p* instead.

In addition, this study highlighted some particularities of the JS4 and JS5 items previously undocumented; both exhibited a significant flooring effect, their interitem correlation was particularly high, and the confirmatory factor analysis model fit was significantly improved when their error terms were covaried. This may indicate that these two items may be measuring the same content of the construct; additional studies are needed to replicate these findings. The current study is also the first to provide insight into the test-retest reliability of the JSPPPE tool; the considerably high values of all ICC coefficients denoted substantial test-retest reliability.

The most striking result to emerge from this study's data is that, except for the TCES personal/emotional subscale, significant negative correlations were revealed between all TCES subscales and the Gr-JSPPPE. To the authors' knowledge, this is the first study attempting to elucidate the association between these two scales. Importantly, this study further substantiates the existing knowledge that inferences regarding physician empathy assessed by self-reported scales do not reflect patient perceptions. Making one step beyond this confirmation, this study denotes that physicians that declare being less empathetic might be perceived by their patients as more empathetic, compared to others. It can be conceivably hypothesized that these physicians may be more likely to underestimate their empathy due to being humbler than others, as empathy and humility seem to be correlated [74].

Further, female sex emerged as a consistent predictor in the TCES cognitive subscales. Indeed, female practitioners seem to express empathy to patients more effectively than their male colleagues, as confirmed by a recent meta-analysis [75]. It has been proposed that the “nurturing investment” theory, suggesting that females have more empathic potential due to offspring raising, may explain this finding [76, 77]. Surprisingly, however, no such evidence was found regarding the TCES emotional subscales; future studies on this topic are therefore required to elucidate these findings. In addition, married and more experienced physicians seemed to declare being more empathic than others at the personal cognitive and professional cognitive level, respectively, corroborating previous work [77]. Again, the reason why no such correlation was noted at the emotional level remains elusive.

Regarding the correlations between quality of life indices and empathy scales, some mixed results emerged. In particular, the negative correlation between the TCES pro/emo subscale and the physicians' EQ-5D index score could not be justified based on existing literature and should be read with caution. On the other hand, the positive correlation between the patients' quality of life and their perception of physician's empathy concurs well with previous research in different settings [12–15, 78, 79].

Some limitations of this study should be acknowledged. Inevitably, the convergent validity was not assessed due to the lack of another validated tool in Greek assessing physicians' empathy by their patients. Furthermore, the small sample size of physicians did not allow for multivariate analyses and may have reduced the overall study's power. Finally, the single-center nature of this study may limit the generalizability of its results.

## 5. Conclusions

This paper evaluated the psychometric properties of the Gr-JSPPPE and found substantial evidence for its validity and reliability, making it the first validated tool to assess patient perceptions of physician empathy in the Greek language. The evidence from this study also intimates that physician empathy assessed by the self-reported scale is inversely associated with patient perceptions. Female sex, marital status, duration of employment in current post, and quality of life emerged as important predictors of physician empathy, while patient quality of life seemed the only predictor of patient perception of physician empathy. Large-scale multicenter studies could shed more light on empathy, both as reported by the physician and as perceived by the patient, in order to improve clinical care outcomes.

## Figures and Tables

**Table 1:** Exploratory factor analysis.

Item code	Item description	Factor loadings	Communalities
JS1	Can view things from my perspective.	0.794	0.631
JS2	Asks about what is happening in my daily life.	0.863	0.745
JS3	Seems concerned about me and my family.	0.859	0.737
JS4	Understands my emotions, feelings, and concerns.	0.805	0.649
JS5	Is an understanding doctor.	0.718	0.515

**Table 2:** Median score values, interquartile ranges, corrected item-total correlations, and corrected Cronbach's alpha values.

Items	Median (IQR)	Item-total correlation^*∗*^	Cronbach's alpha^*∗*^
JS1	6 (3)	0.827	0.921
JS2	5 (3)	0.871	0.913
JS3	5 (3)	0.872	0.912
JS4	7 (2)	0.824	0.922
JS5	7 (1)	0.781	0.931

IQR = interquartile range. ^*∗*^If item deleted.

**Table 3:** Mean values and test-retest reliability using intraclass correlation coefficients.

Items	Test scores	Retest scores	ICC (95% CI)^*∗*^
	Mean (SD)	Mean (SD)	
JS1	5.13 (1.82)	5.83 (1.49)	0.923 (0.846–0.963)^*∗∗*^
JS2	4.71 (1.94)	5.30 (1.92)	0.949 (0.895–0.975)^*∗∗*^
JS3	4.81 (1.91)	5.23 (2.27)	0.863 (0.732–0.932)^*∗∗*^
JS4	5.92 (1.53)	6.17 (1.58)	0.900 (0.801–0.951)^*∗∗*^
JS5	6.12 (1.41)	6.67 (0.80)	0.848 (0.705–0.925)^*∗∗*^
Total	26.69 (7.74)	29.2 (7.03)	0.958 (0.914–0.980)^*∗∗*^

ICC = intraclass correlation coefficients; CI = confidence interval; SD = standard deviation. ^*∗*^ICC values using a two-way mixed-effects model. ^*∗∗*^*p* < 0.001.

**Table 4:** Sociodemographic characteristics of the study sample.

Categorical variables	Group A (*n* = 189)	Group B (*n* = 17)
	*N* (%)	*N* (%)
Sex		
Males	100 (52.9)	8 (47.1)
Females	89 (47.1)	9 (52.9)

Marital status		
Single	23 (12.2)	9 (52.9)
Married/living with partner	117 (61.9)	7 (41.2)
Divorced	13 (6.9)	1 (5.9)
Widowed	36 (19.0)	0 (0.0)

Education		
Illiterate	44 (23.3)	n/a
Primary education	63 (33.3)	n/a
Secondary education	47 (24.9)	n/a
Technological educational institute	14 (7.4)	n/a
University	20 (10.6)	n/a
Postgraduate university education	1 (0.5)	n/a

Professional status		
Employed	33 (17.5)	n/a
Unemployed	16 (8.5)	n/a
Student	8 (4.2)	n/a
Housewife	132 (69.8)	n/a

Net personal monthly income		
O Euros	30 (15.9)	n/a
1–500 Euros	97 (51.3)	n/a
501–1000 Euros	48 (25.4)	n/a
1001–1500 Euros	12 (6.3)	n/a
>1500 Euros	2 (1.1)	n/a

Duration of employment in current post		
0–2 years	n/a	11 (64.7)
3–5 years	n/a	5 (29.4)
6–9 years	n/a	0 (0.0)
10–14 years	n/a	1 (5.9)
≥15 years	n/a	0 (0.0)

Total years of work experience		
0–4 years	n/a	5 (29.4)
5–9 years	n/a	6 (35.3)
10–14 years	n/a	3 (17.6)
≥15 years	n/a	3 (17.6)

Average duration of night sleep		
≤ 5 hours	n/a	10 (58.8)
6–9 hours	n/a	7 (41.2)
≥10 hours	n/a	0 (0.0)
Continuous variables	Mean (SD); median (IQR)	
Age	66 (19.27); 72 (25)	36 (11); 33 (16)
Household members	2 (1.19); 2 (1)	2 (1.00); 2 (2)

SD = standard deviation; IQR = interquartile range; n/a = not applicable.

**Table 5:** Health-related measures.

Group A		
Scales	Mean score (SD)	Median score (IQR)
SAS	29.90 (6.07)	29 (6.5)
PHQ-9	4.37 (3.56)	4 (2)
EQ-5D index score	0.51 (0.35)	0.53 (0.53)
EQ-5D VAS “thermometer”	59.04 (28.49)	65 (50)
VAS pain scale	2.81 (2.92)	2 (6)
Group B		
Scales/subscales	Mean score (SD)	Median score (IQR)
EQ-5D index score	0.51 (0.35)	0.53 (0.53)
EQ-5D VAS “thermometer”	59.04 (28.49)	65 (50)
TCES personal cognitive subscale	46.66 (7.35)	47 (7)
TCES personal emotional subscale	45.88 (7.52)	46 (13)
TCES professional cognitive subscale	47.93 (6.70)	48 (11)
TCES professional emotional subscale	43.55 (9.12)	42 (9.5)

## Data Availability

Data are available upon special request.
